# Comparison of planned versus achieved central corneal stromal thickness reduction in SMILE versus FS-LASIK: a retrospective study

**DOI:** 10.1038/s41598-023-37143-8

**Published:** 2023-06-20

**Authors:** Xueqing Lu, Yimeng Fan, Zhao Liu, Xuanyu Qiu, Qiang Shi, Ning Gao, Shengjian Mi, Cheng Pei

**Affiliations:** Department of Ophthalmology, The First Affiliated Hospital of Xi’an Jiaotong University, 277 West Yanta Road, Xi’an, 710061 Shaanxi China

**Keywords:** Refractive errors, Surgery

## Abstract

Accuracy of planned corneal stromal thickness (CST) reduction is essential to the safety of laser vision correction. This study was to compare the accuracy of the planned central CST reduction in small incision lenticule extraction (SMILE) and femtosecond laser-assisted in situ keratomileusis (FS-LASIK). A total of 77 patients (43 for SMILE, 34 for FS-LASIK using Custom-Q algorithm) were included in this retrospective study. At postoperative 6–18 months, the central CST reduction was overestimated by 18.49 ± 6.42 μm in the SMILE group (P < 0.001) and underestimated by 2.56 ± 7.79 μm in the FS-LASIK group (P = 0.064). The planned-achieved difference (PAD) of central CST reduction was positively correlated with preoperative manifest refraction spherical equivalent (MRSE) and with planned central CST reduction in both groups. When calculated by manifest refraction (MR) without nomogram adjustment, the central CST reduction was overestimated by 11.14 ± 6.53 μm in the SMILE group and underestimated by 2.83 ± 7.39 μm in the FS-LASIK group. The PAD of central CST reduction without nomogram was significantly narrowed in SMILE and maintained in FS-LASIK, suggesting estimation using MR without nomogram adjustment may be feasible for SMILE and FS-LASIK in clinical practice.

## Introduction

Small incision lenticule extraction (SMILE) and femtosecond laser-assisted in situ keratomileusis (FS-LASIK) are two mainstream corneal laser treatment options for myopia with or without astigmatism^1,2^. In SMILE, an intrastromal lenticule is created with femtosecond lasers and taken out through a small incision. FS-LASIK involves excimer laser ablation of corneal stroma beneath a hinged corneal flap^3^.

Many factors affect the accuracy of corneal thickness (CT) reduction in SMILE and FS-LASIK. The patient's preoperative refractive error, consistency of laser energy transfer, duration of the surgery, even temperature and humidity of the operation room may lead to the difference between the achieved and planned CT reduction^4,5^. Further, early postoperative corneal edema and subsequent corneal epithelial^6^ and stromal remodeling after laser vision correction^7^ are also contributing factors. These changes can still be observed even 1 year after surgery^8,9^.

Accuracy of planned CT reduction in SMILE and FS-LASIK attracts more attention now, since it is essential to ensure a safe surgery. Overestimation of central CT reduction could increase the exclusion of eligible patients, while underestimation may increase the risk of iatrogenic corneal dilation and postoperative myopia progression^10,11^. However, the results of previous studies are inconsistent. The CT reduction of SMILE was found overestimated in varying degrees^7,9,12–18^. For FS-LASIK, most studies found the CT reduction was underestimated while a few studies found it overestimated^5,14–20^.

To avoid postoperative refractive error and improve visual outcomes, surgeons designed nomograms. The nomogram is a series of formulas that appropriately adjust a patient's manifest refraction (MR) before we input it into the laser portal software^21–23^. Previous studies used surgical parameters with nomogram adjustment to evaluate the accuracy of planned CT reduction, which inherently ignored the influence of nomogram^15–17,24^.

In this retrospective study, we measured the thickness of the cornea and stroma before and after the surgery; and evaluated the predictability of central corneal stromal thickness (CST) reduction during postoperative 6–18 months in both SMILE and FS-LASIK. We further investigated the effect of nomogram adjustment on the accuracy of estimation. Together, this study shed a light on future researches to improve the prediction of planned central CST reduction.

## Methods

### Design and patients

This retrospective study involved a total of 77 eyes of 77 consecutive patients whose follow-up results during postoperative 6–18 months were recorded. All enrolled patients underwent laser vision correction for myopia or myopia with astigmatism in the Ophthalmology Refractive Surgery Center of the First Affiliated Hospital of Xi'an Jiaotong University from April 2019 to October 2021. Forty-three eyes of 43 patients underwent SMILE surgery, and 34 eyes of 34 patients underwent FS-LASIK surgery. According to preoperative manifest refraction spherical equivalent (MRSE), all enrolled patients were recorded into established subgroups, which included mild myopia subgroup (MRSE < − 3 D, 21 patients), moderate myopia subgroup (− 3 D ≤ MRSE < − 6 D, 26 patients), and high myopia subgroup (− 6 D ≤ MRSE < − 8 D, 30 patients).

Inclusion criteria: (1) age ≥ 18 years and ≤ 37 years; (2) preoperative best-corrected distance visual acuity (CDVA) ≥ 1.0; (3) sphere up to − 8 D; (4) cylinder < − 2 D; (5) a refractive stability in the past year, with an increase of ≤ 0.5 D; (6) discontinued soft contact lenses wearing for more than 1 week, and discontinued orthokeratology lenses wearing for more than 3 months; (7) predicted thickness of the residual stromal bed at the thinnest point in the central cornea was more than 280 μm after lenticules removal or stromal ablation.

Exclusion criteria: (1) complicated with severe diabetes, systemic connective tissue diseases, or autoimmune diseases; (2) active eye diseases; (3) suspected and confirmed topographic evidence of corneal ectasia; (4) previous history of corneal refractive surgeries or eye surgeries; (5) women during pregnancy or lactation; (6) no complications affecting vision were observed during 6–18 months follow-up period.

The study was conducted in accordance with the Declaration of Helsinki and was approved by the Medical Ethics Committee of First Affiliated Hospital of Xi’an Jiaotong University (2019-W20). Written informed consent was obtained from each patient before the surgical procedure.

### Surgical technique

All enrolled patients underwent a comprehensive eye examination before surgeries to exclude operative contraindications. Routine application of 0.5% levofloxacin (Santen Pharmaceutical Co., Ltd., Osaka, Japan) 12 times was performed 1–3 days before surgeries. The same senior physician (SM) performed all surgeries under topical anesthesia with postoperative target refraction of 0 D. All enrolled patients cooperated well during the surgery, and all surgeries were successful without intraoperative accident or complication.

SMILE was performed by the VisuMax^®^ femtosecond laser system (Carl Zeiss Meditec AG, Jena, Germany). In all cases, a 2-mm width incision with 90° of side-out angle was created at 120° of the cornea. The cap thickness was 120 µm, and the diameter was 6.8–7.3 mm. The lenticule optical zone was 6.0–6.5 mm. The minimum lenticule thickness was set at 15 µm. The nomogram was adjusted by 0.5 D overcorrection of spherical refraction when the MR sphere ≤ − 5 D and 10% more spherical refraction adding when the MR sphere > − 5 D.

For FS-LASIK, all flaps were created by the WaveLight^®^ FS200 femtosecond laser (Alcon Management S. A., Fort Worth, United States). The flap thickness was 100–110 µm; the side-cut angle was 105°. Following blunt dissection and flap lift, the stromal bed was ablated with the WaveLight^®^ EX500 excimer laser (Custom-Q algorithm, Alcon Management S. A., Fort Worth, United States) using an optic zone of 6.0–6.5 mm with a 1.25 mm transition zone. The nomogram used to adjust the spherical degree is shown in Supplementary Table S1. The corneal Q values within the 6 mm diameter area measured by SIRIUS tomograph and corneal topographer (CSO Ophthalmic, Scandicci, Italy) was recorded as the postoperative target.

### Postoperative care

For all patients, postoperative medications were routinely prescribed as follows: 0.5% levofloxacin four times a day for a week; 0.1% fluorometholone (Santen Pharmaceutical Co., Ltd., Osaka, Japan) for 40 days, four times a day, reduced once every 10 days; artificial tears four times a day, 1–6 months as appropriate.

### Calculation and record of main outcomes

The following data were recorded before surgeries and at the first follow-up during 6–18 months period after surgeries: patient demographics, clinical signs (examined with slit lamp microscope), preoperative best CDVA, and postoperative uncorrected distance visual acuity (UDVA). We also recorded intraocular pressure, refraction measured by Auto Refractometer AR-1 (NIDEK CO., LTD. Aichi, Japan), and CT measured by RTVue XR 100 Optical Coherence Tomography (Optovue Corporate, Fremont, United States) before and after the surgeries. Three measurements were taken each time, with central corneal epithelial thickness (CET) differences of no more than 1 µm. Mean CT and CET were recorded within the central 2 mm diameter zone of the cornea. All dominant eye data were selected for analysis.

The software installed in the refractive laser platforms provided the planned central CST reduction. We define the planned-achieved difference (PAD) as the difference between planned and achieved central CST reduction. Considering that corneal epithelium remodeling usually occurs after laser vision correction, it is necessary to exclude the effect of postoperative corneal epithelial hyperplasia. The calculated formula for the achieved central CT reduction was central CT_pre_–central CT_post_, and the achieved central CST reduction was (central CT_pre_–central CET_pre_)–(central CT_post_–central CET_post_). We also recorded the central CST reduction predicted by laser platform software without nomogram adjustment, while other parameters remained consistent. The efficacy index (EI) was calculated as Postoperative UDVA/Preoperative CDVA.

### Statistical analysis

All statistical analyses were performed using SPSS 25.0 for Windows (IBM, Armonk, United States). The normality of all data samples was assessed with the Kolmogorov–Smirnov test. The paired sample t-test and one-way variance (ANOVA) were used to compare normally distributed variables. Post-hoc multiple comparisons were performed between groups using Dunnett’s T. Wilcoxon signed rank test and the Kruskal–Wallis H test were used to compare the non-normally distributed data, and post-hoc pairwise comparisons were performed using the Dunn–Bonferroni test. The Chi-square test was used for comparisons between categorical variable groups. The linear regression analysis was performed to explore the linear relationship between variables. The level of statistical significance was set at a P value of less than 0.05. Visual acuity was converted into the logMAR scale from the decimal notation for statistical analysis.

## Results

### Baseline and visual outcomes

There were no significant differences in patient demographics and other primary baseline data between SMILE and FS-LASIK groups (Table 1). When performing subgroup analyses, the preoperative sphere and MRSE in the mild myopia subgroup of SMILE were higher than those in FS-LASIK (Supplementary Table S2).

**Table 1:** Demographics and clinical characteristics at baseline.

Characteristics	SMILE (n = 43)	FS-LASIK (n = 34)	*P* value
Age (years)	25.12 ± 5.09	27.56 ± 6.01	0.057
Male, n (%)	15 (34.88%)	14 (41.12%)	0.571
Right eye, n (%)	21 (48.84%)	20 (58.82)	0.383
CDVA (logMAR)	− 0.06 ± 0.04	− 0.07 ± 0.05	0.812
Preoperative sphere (D)	− 4.66 ± 1.68	− 4.44 ± 2.09	0.773
Preoperative cylinder (D)	− 0.61 ± 0.48	− 0.62 ± 0.51	0.954
Preoperative MRSE (D)	− 4.96 ± 1.71	− 4.74 ± 2.15	0.739
Central CT (μm)	537.23 ± 22.33	517.80 ± 26.25	0.001
Postoperative UDVA (logMAR)	− 0.08 ± 0.13	− 0.09 ± 0.07	0.749
Postoperative refraction* (D)	− 0.32 ± 0.52	− 0.41 ± 0.55	0.456
EI	1.06 ± 0.24	1.07 ± 0.17	0.974

The values are presented as mean ± standard deviation unless otherwise noted. Statistically significant at P ≤ 0.05. *Measured by auto refractometer.

*SMILE* Small incision lenticule extraction. *FS-LASIK* femtosecond laser-assisted in situ keratomileusis. *CDVA* corrected distance visual acuity. *UDVA* uncorrected distance visual acuity. *MRSE* manifest refraction spherical equivalent. *CT* corneal thickness. *EI* efficacy index. *D* diopter.

**Figure 1:**
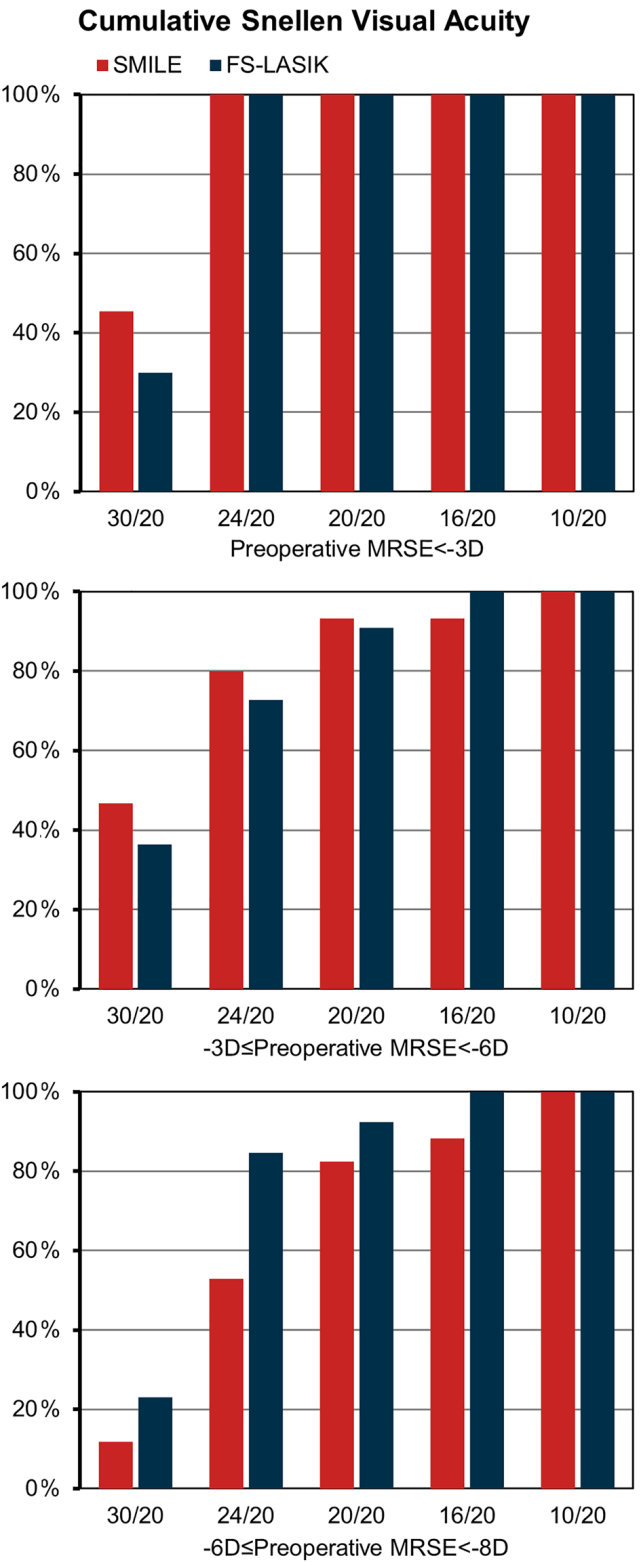
Postoperative UDVA in SMILE and FS-LASIK. *UDVA* uncorrected distance visual acuity. *SMILE* small-incision lenticule extraction. *FS-LASIK* femtosecond laser-assisted in situ keratomileusis. *MRSE* manifest refraction spherical equivalent. *D* diopter.

Postoperative visual outcomes are shown in Fig. 1. All enrolled patients achieved good UDVA. In the mild subgroup, a UDVA of 20/20 or better was measured in all of the eyes in both groups. In the moderate subgroup, a UDVA of 20/20 or better was measured in 93.33% (14/15) of eyes in SMILE and 90.91% (10/11) in FS-LASIK. In the high subgroup, a UDVA of 20/20 or better was measured in 82.35% (14/17) of eyes in SMILE and 92.31% (12/13) in FS-LASIK. No significant difference in postoperative UDVA between the two groups was observed (− 0.08 ± 0.13 vs. − 0.09 ± 0.07, P = 0.456, Table 1). The EI reached 1.06 ± 0.24 in SMILE and 1.07 ± 0.17 in FS-LASIK, no statistical difference was found between the two groups (P = 0.974, Table 1).

### Predictability of central CST reduction with nomogram adjustment

For the overall achieved central CST reduction, no statistically significant difference was found between the SMILE and FS-LASIK groups (78.26 ± 16.58 μm vs. 72.44 ± 29.13 μm, P = 0.466, Table 2). However, the more achieved central CST reduction was found in the mild subgroup of SMILE (57.09 ± 5.41 μm vs. 35.90 ± 9.66 μm, P < 0.001, Table 2), while no significant difference was found in the other two subgroups (P = 0.678 and P = 0.054, Table 2).

**Table 2:** Planned and achieved central CST reduction of SMILE and FS-LASIK.

Characteristic	All eyes			Mild (MRSE < − 3D)			Moderate (− 3D ≤ MRSE < − 6D)			High (− 6D ≤ MRSE < − 8D)		
	SMILE (n = 43)	FS-LASIK (n = 34)	P*	SMILE (n = 11)	FS-LASIK (n = 10)	P*	SMILE (n = 15)	FS-LASIK (n = 11)	P*	SMILE (n = 17)	FS-LASIK (n = 13)	P*
Planned central CST reduction with nomogram (μm)	96.74 ± 19.71	69.89 ± 25.24	< 0.001	69.91 ± 5.68	37.10 ± 10.05	< 0.001	95.73 ± 11.39	71.10 ± 10.17	< 0.001	115.00 ± 6.02	94.08 ± 7.68	< 0.001
Achieved central CST reduction (μm)	78.26 ± 16.58	72.44 ± 29.13	0.466	57.09 ± 5.41	35.90 ± 9.66	< 0.001	77.33 ± 12.08	73.73 ± 16.52	0.678	92.77 ± 6.60	99.46 ± 11.48	0.054
P**	< 0.001	0.069	–	< 0.001	0.257	–	< 0.001	0.155	–	< 0.001	0.073	–

The values are presented as mean ± standard deviation. Statistically significant at P ≤ 0.05.

*SMILE* small incision lenticule extraction, *FS-LASIK* femtosecond laser-assisted in situ keratomileusis, *CST* corneal stromal thickness, *D* diopter.

*Comparison between SMILE and FS-LASIK. **Comparison between planned and achieved central CST reduction.

**Figure 2:**
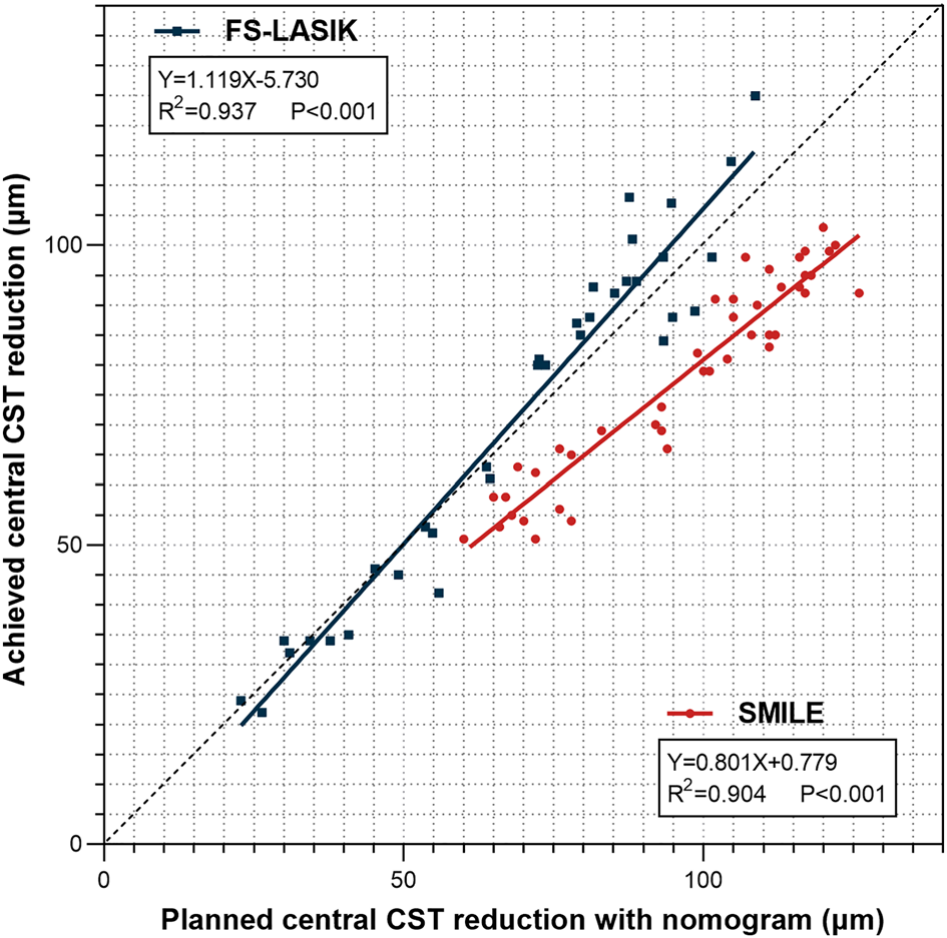
Comparison of the planned and achieved central CST reduction with nomogram. The coefficients of determination (R^2^) and regression equations are displayed. Points plotted above the dotted lines indicate that the planned central CST reduction underestimates the achieved central CST reduction and points plotted below the dotted lines indicate that the planned central CST reduction overestimates the achieved central CST reduction. *SMILE* small-incision lenticule extraction. *FS-LASIK* femtosecond laser-assisted in situ keratomileusis. *CST* corneal stromal thickness.

**Figure 3:**
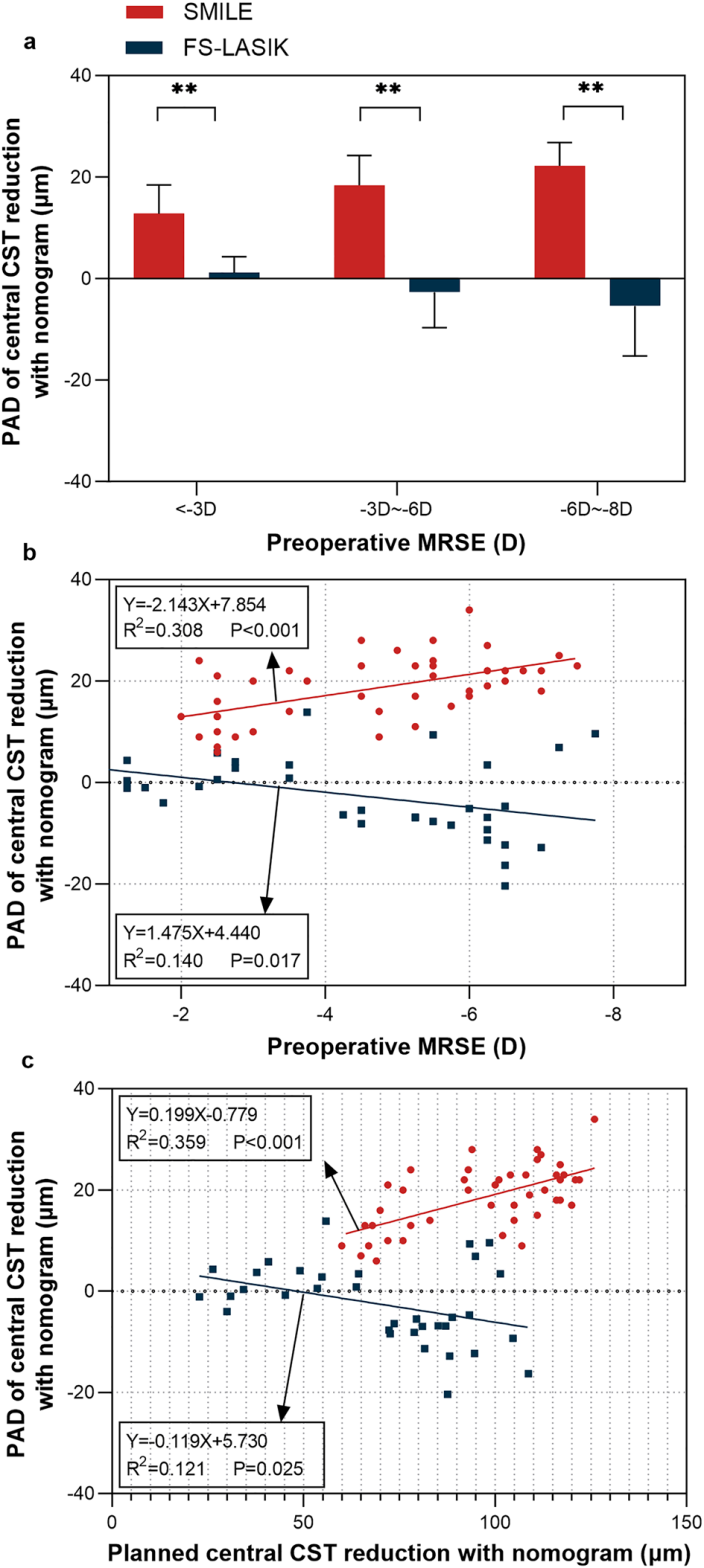
Analysis and comparison for PAD of central CST reduction with nomogram adjustment. *SMILE* small-incision lenticule extraction. *FS-LASIK* femtosecond laser-assisted in situ keratomileusis. *MRSE* manifest refraction spherical equivalent. *PAD* planned-achieved difference. *D* diopter. **Statistically significant at P ≤ 0.001.

Next, we compared the difference between planned and achieved central CST reduction which we defined as the planned-achieved difference (PAD). Here, the planned central CST reduction was estimated with nomogram adjustment, which is consistent with clinical practice. Close correlations between the planned and achieved central CST reduction were observed in both groups (P < 0.001, Fig. 2). The R^2^ values of SMILE and FS-LASIK were 0.904 and 0.937, respectively. SMILE displayed a significant difference between the planned and achieved central CST reduction (96.74 ± 19.71 μm vs. 78.26 ± 16.58 μm, P < 0.001, Table 2), while no significant difference was found in FS-LASIK (69.89 ± 25.24 μm vs. 72.44 ± 29.13 μm, P = 0.064, Table 2). The PAD was 18.49 ± 6.42 µm in the SMILE group and − 2.56 ± 7.79 µm in the FS-LASIK group. The PAD of SMILE in all three subgroups was higher than FS-LASIK (All P < 0.001, Fig. 3a). The linear regression analyses indicated that PAD was correlated with preoperative MRSE (Fig. 3b) and the planned central CST reduction with nomogram (Fig. 3c) in both groups. Neither postoperative UDVA nor EI was statistically correlated with the PAD.

### PAD of central CST reduction without nomogram adjustment

We wondered whether the planned central CST reduction estimated without nomogram adjustment was more helpful for preoperative planning, a review was carried out. We conducted a further analysis using the planned central CST reduction output from laser platform software without nomogram adjustment.

**Figure 4:**
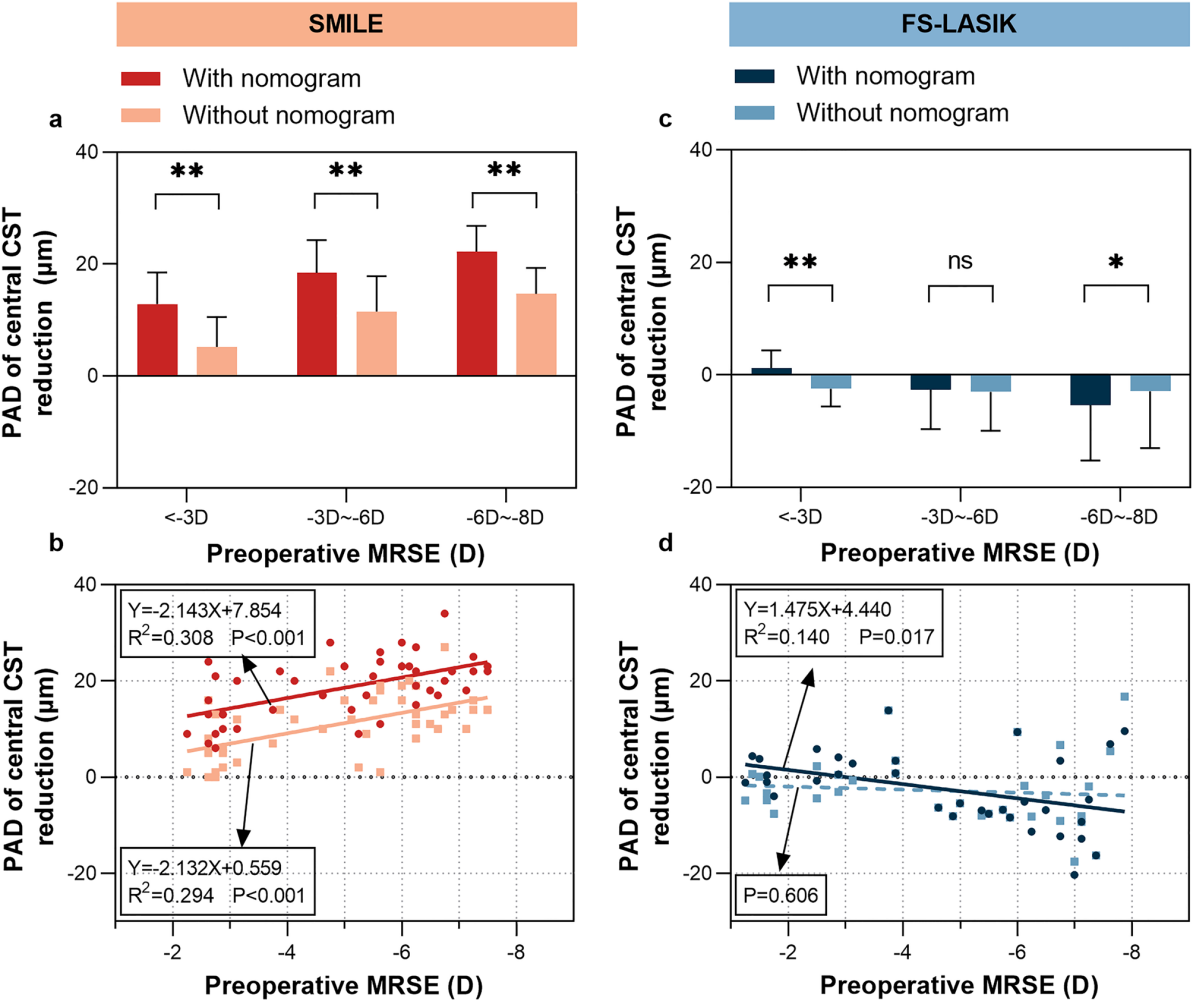
Analysis and comparison for PAD of central CST reduction without nomogram adjustment. *SMILE* small-incision lenticule extraction. *FS-LASIK* femtosecond laser-assisted in situ keratomileusis. *MRSE* manifest refraction spherical equivalent. *PAD* planned-achieved difference. *D* diopter. **Statistically significant at P ≤ 0.001. *Statistically significant at P ≤ 0.05. ns: Statistically significant at P > 0.05.

In the SMILE group, the PAD of central CST reduction without nomogram adjustment was narrowed to 11.14 ± 6.53 μm when compared to that with nomogram (11.14 ± 6.53 μm vs. 18.49 ± 6.42 μm, P < 0.001, Table 3), same trends were found in all three subgroups (All P < 0.001, Table 3, Fig. 4a). The PAD was positively correlated with preoperative MRSE (R^2^ = 0.294, P < 0.001, Fig. 4b) and the planned central CST reduction without nomogram adjustment (R^2^ = 0.357, P < 0.001), which was similar to the estimation with nomogram adjustment.

**Table 3:** Comparison of PAD of central CST/CT reduction with or without nomogram.

Characteristic	All eyes		Mild (MRSE < − 3D)		Moderate (− 3D ≤ MRSE < − 6D)		High (− 6D ≤ MRSE < − 8D)	
	SMILE (n = 43)	FS-LASIK (n = 34)	SMILE (n = 11)	FS-LASIK (n = 10)	SMILE (n = 15)	FS-LASIK (n = 11)	SMILE (n = 17)	FS-LASIK (n = 13)
PAD of central CST reduction with nomogram (μm)	18.49 ± 6.42	− 2.56 ± 7.79	12.82 ± 5.65	1.20 ± 3.14	18.40 ± 5.88	− 2.63 ± 7.05	22.24 ± 4.58	− 5.39 ± 9.88
PAD of central CST reduction without nomogram (μm)	11.14 ± 6.53	− 2.83 ± 7.39	5.18 ± 5.34	− 2.48 ± 3.16	11.53 ± 6.31	− 3.04 ± 6.91	14.65 ± 4.64	− 2.93 ± 10.13
P*	< 0.001	0.246	< 0.001	< 0.001	< 0.001	0.137	< 0.001	0.002
PAD of central CT reduction with nomogram (μm)	23.02 ± 7.87	1.91 ± 7.53	14.55 ± 4.72	2.60 ± 3.39	23.00 ± 6.08	1.73 ± 6.66	28.53 ± 5.90	1.53 ± 10.48
PAD of central CT reduction without nomogram (μm)	15.76 ± 8.04	1.64 ± 7.67	6.90 ± 4.41	− 1.08 ± 3.42	16.13 ± 6.95	1.32 ± 6.88	20.94 ± 5.74	4.00 ± 10.07
P**	< 0.001	0.590	< 0.001	< 0.001	< 0.001	0.109	< 0.001	0.002

The values are presented as mean ± standard deviation. Statistically significant at P ≤ 0.05.

*SMILE* small incision lenticule extraction, *FS-LASIK* femtosecond laser-assisted in situ keratomileusis, *CT* corneal thickness, *CST* corneal stromal thickness, *PAD* planned-achieved difference, *D* diopter.

*Comparison between PAD of central CST reduction with or without nomogram. **Comparison between PAD of central CT reduction with or without nomogram.

In the FS-LASIK group, the PAD of central CST reduction without nomogram adjustment was − 2.83 ± 7.39 μm, which was not statistically significantly different from the PAD with nomogram adjustment (− 2.83 ± 7.39 μm vs. − 2.56 ± 7.79 μm, P = 0.246, Table 3). In the subgroup analyses, there were significant differences in mild and high subgroups (P < 0.001 and P = 0.002, respectively, Table 3, Fig. 4c), while no significant difference was observed in the moderate group (P = 0.137, Table 3, Fig. 4c). Unlike the PAD with nomogram adjustment, the PAD without nomogram in FS-LASIK was not significantly correlated with preoperative MRSE (P = 0.606, Fig. 4d) nor the planned central CST reduction (P = 0.625).

### PAD of central CT reduction with or without nomogram adjustment

Considering measurement of the full corneal thickness is more commonly used for preoperative planning in clinical practice, a further analysis of central CT reduction was performed. The results were similar to central CST reduction (Table 3).

In the SMILE group, the PAD of central CT reduction without nomogram adjustment was smaller than that with nomogram adjustment (23.02 ± 7.87 μm vs. 15.76 ± 8.04 μm, P < 0.001, Table 3). Similar trends were found in all three subgroups (All P < 0.001, Table 3). In the FS-LASIK group, no significant difference was observed between the PAD of central CT reduction with nomogram adjustment and that without nomogram (1.91 ± 7.53 μm vs. 1.64 ± 7.67 μm, P = 0.590, Table 3), while the differences were statistically significant in some subgroups (P < 0.001 for mild subgroup, P = 0.109 for moderate subgroup, and P = 0.002 for high subgroup, Table 3).

Since the PAD of central CT reduction included the remodeling of corneal epithelium, we also analyzed the postoperative central CET increases, which were obtained using the postoperative central CET to subtract the preoperative central CET. When comparing the increases of CET between SMILE and FS-LASIK, no significant difference was observed either by overall analysis (4.54 ± 4.66 μm vs. 4.47 ± 5.36 μm, P = 0.947, Table 4) or by subgroup analyses (All P > 0.05, Table 4). These results may explain why the PAD of central CT reduction was similar to that of central CST reduction.

**Table 4:** Increases of CET after SMILE and FS-LASIK.

Characteristic	All eyes			Mild (MRSE < − 3D)			Moderate (− 3D ≤ MRSE < − 6D)			High (− 6D ≤ MRSE < − 8D)		
	SMILE (n = 43)	FS-LASIK (n = 34)	P	SMILE (n = 11)	FS-LASIK (n = 10)	P	SMILE (n = 15)	FS-LASIK (n = 11)	P	SMILE (n = 17)	FS-LASIK (n = 13)	P
Central CET increases (μm)	4.54 ± 4.66	4.47 ± 5.36	0.947	1.73 ± 1.90	1.40 ± 3.17	0.775	4.60 ± 3.25	4.36 ± 3.23	0.856	6.29 ± 6.06	6.92 ± 6.96	0.794
Achieved central CST reduction (μm)	78.26 ± 16.58	72.44 ± 29.13	0.466	57.09 ± 5.41	35.90 ± 9.66	< 0.001	77.33 ± 12.08	73.73 ± 16.52	0.678	92.77 ± 6.60	99.46 ± 11.48	0.054
Achieved central CT reduction (μm)	73.72 ± 14.95	67.97 ± 27.09	0.272	55.36 ± 4.11	34.50 ± 10.30	< 0.001	72.73 ± 11.32	69.36 ± 14.96	0.721	86.47 ± 7.69	92.54 ± 12.77	0.117

The values are presented as mean ± standard deviation. Statistically significant at P ≤ 0.05.

*SMILE* small incision lenticule extraction, *FS-LASIK* femtosecond laser-assisted in situ keratomileusis, *CET* corneal epithelial thickness, *CST* corneal stromal thickness, *CT* corneal thickness, *D* diopter.

## Discussion

Accurate estimation of central CST reduction is essential in laser vision correction. In this study, we found the achieved central CST reduction was highly correlated with the planned central CST reduction for both SMILE and FS-LASIK, while FS-LASIK showed an even closer estimation than SMILE. With respect to the influence of nomogram adjustment, we further found the PAD of central CST reduction without nomogram was significantly narrowed in SMILE and maintained in FS-LASIK. In addition, we found that the achieved full CT reduction was parallel to the achieved CST reduction, suggesting the full CT reduction can also be used for estimation.

For SMILE, central CST reduction was overestimated by 18.49 ± 6.42 μm with nomogram adjustment. The PAD of central CST reduction was positively related to the planned central CST reduction as well as preoperative MRSE, which means poor predictability of thickness reduction volume was observed when planned central CST reduction and preoperative MRSE increased. There are some hypotheses to explain this phenomenon. First, removing partial collagen lamellae of the cornea could cause stromal expansion after surgery through the mechanism of stress release and biomechanical remodeling. Such a stromal expansion may partially offset the central stromal thickness reduction and lead to the deviation^7,14^. Higher refractive correction means thicker lenticules removal, which could lead to more prominent tension release and stromal expansion, just as revealed in our study. Second, keratocyte-mediated wound healing in the laser-cut interface might be another source of postoperative stromal thickening^7^.

Considering the effect of nomogram adjustment on the accuracy of estimation, we further calculated the PAD of central CST reduction based on MR without nomogram adjustment. It was 11.14 ± 6.53 μm in SMILE, which was significantly narrowed when compared to the PAD with nomogram adjustment in both overall and subgroup analyses. This finding indicates that MR can be directly used to estimate the central CST reduction. It is noted that the estimation of central CST reduction without nomogram adjustment was still overestimated, suggesting that it is still safe for SMILE. The possible reason is that the nomogram can cause overestimation. The larger nomogram induced by the higher MR, the more overestimation occurred (e.g., − 8.00 D will be programmed as − 8.80 D). Removing the nomogram, the planned central CST reduction calculated by MR is closer to the postoperative achieved central CST reduction. It should be noted that, even removing the nomogram, the PAD of central CST reduction was still correlated with planned central CST reduction and preoperative MRSE, indicating that these two factors still influence the accuracy of prediction.

For FS-LASIK, the PAD of central CST reduction with nomogram adjustment was − 2.56 ± 7.79 μm. The postoperative achieved central CST reduction was consistent with preoperative predictions (72.44 ± 29.13 μm vs. 69.89 ± 25.24 μm, P = 0.466, Table 2). Similar results of postoperative achieves consistent with preoperative predictions were also found in the three subgroups (all P > 0.05, Table 2). Meanwhile, we found the PAD of central CST reduction with nomogram adjustment was correlated with the planned central CST reduction and preoperative MRSE. Previous studies showed inconsistent results, while overcorrection was found in most studies^15,17,19,20^. Among these studies, it was speculated that such overcorrection might be blamed on the intraoperative laser ablation error^19^. A higher volume of stromal ablation leads to longer surgery duration, and the cornea will be more dehydrated. Meanwhile, the intraoperative central cornea flattens which reduces the cosine effect. These two factors both improve the efficiency of laser ablation and lead to overcorrection^16,19^. As the patient’s preoperative MRSE rises and the duration of surgery increases, the overcorrection becomes more pronounced, which is determined by the laser ablation principle of FS-LASIK^25^. However, no such overcorrection was observed in our study. It is worth noticing that we did not include patients with ultra-high myopia (MRSE ≥ − 8 D), who may face higher overcorrection based on the hypothesis and were at high risk for iatrogenic corneal dilation.

Removing nomogram, the PAD of central CST reduction was maintained in FS-LASIK (− 2.56 ± 7.79 μm vs. − 2.83 ± 7.39 μm, P = 0.246, Table 3). Interestingly, when calculated with nomogram, the PAD of central CST reduction showed a trend of under-correction in the mild subgroup (1.20 ± 3.14 μm, Table 3) and overcorrection in the high subgroup (− 5.39 ± 9.88 μm, Table 3), although it was not statistically significant. No such trend was observed after removing the nomogram (− 2.48 ± 3.16 μm, − 3.04 ± 6.91 μm and − 2.93 ± 10.13 μm respectively for three subgroups). It is probably because the nomogram adjustment we used for FS-LASIK overestimated the CST reduction in mild myopia and underestimated it in high myopia, offsetting part of under-correction and overcorrection. Furthermore, the PAD of central CST reduction without nomogram adjustment was not affected by parameters such as planned central CST reduction and preoperative MRSE. Based on these results, the estimation calculated without nomogram adjustment may provide a more reliable prediction for FS-LASIK.

A noteworthy finding was no significant difference was found in achieved central CST reduction between SMILE and FS-LASIK (78.26 ± 16.58 μm vs. 72.44 ± 29.13 μm, P = 0.466, Table 2). However, the subgroup analyses revealed a higher central CST reduction in SMILE than FS-LASIK in the mild myopia subgroup. The possible reason might be that the preoperative MRSE of mild subgroup was greater in the SMILE group than that in the FS-LASIK group (Supplementary Table S2).

In this study, we found the PAD of central CST reduction was not correlated with postoperative UDVA nor EI in both SMILE or FS-LASIK groups. This finding is in agreement with most of the previous studies^7,17^. Nevertheless, Wang et al. proposed inconsistent results for SMILE, finding that under-correction of achieved central CST reduction in SMILE might trigger slight myopia regression^13^.

Postoperative epithelial hyperplasia increases the postoperative corneal thickness. Thus, calculating the corneal stromal reduction by subtracting the postoperative corneal thickness measurement from the preoperative measurement, the stromal ablation depth will be underestimated. However, due to the limitation of the diagnostic devices, some studies did not exclude the influence of epithelial hyperplasia^16,20,24^ or set a constant for epithelial hyperplasia thickness to correct this bias^5^. In this study, we evaluated the thickness of the corneal epithelium and stroma separately, through which we can exclude the interference of postoperative epithelial hyperplasia thickness, thus avoiding the underestimation of the achieved central CST reduction. We found central CET increases were consistent between SMILE and FS-LASIK across all three refractive ranges. Besides, the achieved full CT reduction was parallel to the achieved CST reduction, suggesting the full CT reduction can be used for estimation, especially for refractive surgery centers that lack of device to separately measure the corneal stroma thickness.

There are several limitations to this study. First, this study is a retrospective study based on patient records. The postoperative MR results were not routinely recorded in our center and therefore they were not included in this study. In addition, due to the lack of early postoperative measurement records, intraoperative CST cutting volume cannot be differentiated from postoperative stromal expansion and rebranding. Second, we did not include patients with MRSE ≥ − 8.00 D, which may have caused bias. Third, we did not stratify and match enrolled patients according to optical zones, nor did we standardize the minimum lenticule thickness. Although our subgroup studies have met the required minimum sample size^14,17^, further validation from prospective studies with larger sample sizes, broader range of age and refraction errors, and extended follow-up period should be performed to observe the long-term progression of achieved epithelial and stromal thickness reductions.

## Conclusions

In conclusion, SMILE and FS-LASIK provided similar visual outcomes during postoperative 6–18 months. The achieved central CST reduction was highly correlated with the planned central CST reduction for both SMILE and FS-LASIK, while FS-LASIK showed an even closer estimation than SMILE. When removing nomogram adjustment, the PAD of central CST reduction was significantly narrowed in SMILE and maintained in FS-LASIK, suggesting estimation using MR without nomogram adjustment may be feasible in practice. Furthermore, the full CT reduction can also be used for estimation.

## Supplementary Information

Supplementary Tables.

## Data Availability

The datasets used and/or analyzed during the current study are available from the corresponding author on reasonable request.
