# Supplementary material for: Comparison of planned versus achieved central corneal stromal thickness reduction in SMILE versus FS-LASIK: a retrospective study
Source: Sci Rep. 2023 Jun 20;13:9956. doi: 10.1038/s41598-023-37143-8 (PMC10281974; doi:10.1038/s41598-023-37143-8)
Supplement: Supplementary file 1 — Supplementary Tables. [file 41598_2023_37143_MOESM1_ESM.pdf]

Comparison of Planned Versus Achieved Central Corneal Stromal Thickness Reduction in SMILE Versus FS-LASIK: a Retrospective Study

Xueqing Lu[1,¶], Yimeng Fan[1,¶], Zhao Liu[1,*], Xuanyu Qiu[1], Qiang Shi[1], Ning Gao[1], Shengjian Mi[1], Cheng Pei[1]

[1]Department of Ophthalmology, the First Affiliated Hospital of Xi'an Jiaotong University, Xi'an, Shaanxi 710061, China

¶ These authors contributed equally to this work.

*Correspondence to: Zhao Liu, M.D., Ph.D. Department of Ophthalmology, the First Affiliated Hospital of Xi'an Jiaotong University, 277 West Yanta Road, Xi'an, Shaanxi 710061, China. Tel: (86) 029-85324452, dr.liuzhao@foxmail.com

Supplementary information

Supplementary Table S1. Nomogram used to adjust the spherical degree for FS-LASIK

Manifest spherical refraction	Nomogram adjustment	Subtract value
-1.00	-1.25	0.25
-1.25	-1.50	0.25
-1.50	-1.75	0.25
-1.75	-2.00	0.25
-2.00	-2.25	0.25
-2.25	-2.50	0.25
-2.50	-2.75	0.25
-2.75	-3.00	0.25
-3.00	-3.00	0
-3.25	-3.25	0
-3.50	-3.50	0
-3.75	-3.75	0
-4.00	-4.00	0
-4.25	-4.25	0
-4.50	-4.50	0
-4.75	-4.75	0
-5.00	-5.00	0
-5.25	-5.25	0
-5.50	-5.50	0
-5.75	-5.75	0
-6.00	-5.75	-0.25
-6.25	-6.00	-0.25
-6.50	-6.20	-0.30
-6.75	-6.40	-0.35
-7.00	-6.60	-0.40
-7.25	-6.75	-0.50
-7.50	-6.95	-0.55
-7.75	-7.15	-0.60
-8.00	-7.30	-0.70

Supplementary Table S2. Demographics and clinical characteristics of subgroups at baseline

Characteristic	Mild (MRSE < -3 D)			Moderate -3 D ≤ MRSE < -6 D)			High (-6 D ≤ MRSE < -8 D)		
	SMILE (n = 11)	FS-LASIK (n = 10)	p	SMILE (n = 15)	FS-LASIK (n = 11)	p	SMILE (n = 17)	FS-LASIK (n = 13)	p
**Age (years)**	25.46 ± 6.42	25.30 ± 7.33	0.959	24.33 ± 4.72	27.82 ± 5.98	0.109	25.59 ± 4.66	29.08 ± 4.72	0.053
**Male, n (%)**	5 (45.45%)	7 (70.00%)	0.387	4 (26.67%)	4 (36.36%)	0.683	6 (35.29%)	3 (23.08%)	0.469
**Right eye, n (%)**	4 (36.36%)	4 (40.00%)	1.000	7 (46.67%)	7 (63.63%)	0.453	10 (58.82%)	9 (69.23%)	0.558
**CDVA (logMAR)**	1.21 ± 0.11	1.22 ± 0.17	1.000	1.12 ± 0.10	1.16 ± 0.08	0.243	1.17 ± 0.08	1.14 ± 0.10	0.408
**Preoperative sphere (D)**	-2.43 ± 0.20	-1.78 ± 0.68	0.030	-4.42 ± 0.86	-4.41 ± 0.96	0.983	-6.31 ± 0.64	-6.50 ± 0.57	0.362
**Preoperative cylinder (D)**	-0.52 ± 0.49	-0.43 ± 0.47	0.649	-0.57 ± 0.36	-0.57 ± 0.37	0.936	-0.71 ± 0.57	-0.81 ± 0.59	0.654
**Preoperative MRSE (D)**	-2.69 ± 0.18	-1.99 ± 0.63	0.015	-4.70 ± 0.89	-4.69 ± 0.92	0.985	-6.66 ± 0.52	-6.90 ± 0.58	0.235
**Central CT (μm)**	528.55 ± 18.73	521.30 ± 27.89	0.489	532.40 ± 21.51	507.18 ± 22.79	0.008	547.19 ± 22.51	524.08 ± 26.88	0.016
**Postoperative UDVA (logMAR)**	1.38 ± 0.25	1.29 ± 0.15	0.409	1.30 ± 0.34	1.27 ± 0.24	0.600	1.08 ± 0.27	1.23 ± 0.18	0.087
**Postoperative refraction* (D)**	-0.01 ± 0.44	-0.19 ± 0.59	0.429	-0.27 ± 0.47	-0.47 ± 0.45	0.284	-0.56 ± 0.53	-0.53 ± 0.58	0.867
**Efficacy index**	1.14 ± 0.14	1.07 ± 0.15	0.241	1.16 ± 0.27	1.06 ± 0.18	0.316	0.92 ± 0.21	1.09 ± 0.19	0.041
**Optical zone diameter (mm), n (range)**	6.5 (6.0-6.5)	6.5 (6.5-6.5)	0.039	6.3 (6.0-6.5)	6.5 (6.0-6.5)	0.069	6.1 (6.0-6.3)	6.3 (6.0-6.5)	0.011

The values are presented as mean ± standard deviation unless otherwise noted. Statistically significant at P ≤ 0.05. *: Measured by auto refractometer. *SMILE* Small incision lenticule extraction. *FS-LASIK* femtosecond laser-assisted in situ keratomileusis. *CDVA* corrected distance visual acuity. *UDVA* uncorrected distance visual acuity. *MRSE* manifest refraction spherical equivalent. *CT* corneal thickness. *D* diopter.
